# Toddlers Favor Communicatively Presented Information over Statistical Reliability in Learning about Artifacts

**DOI:** 10.1371/journal.pone.0122129

**Published:** 2015-03-17

**Authors:** Hanna Marno, Gergely Csibra

**Affiliations:** 1 Language, Cognition and Development Lab, SISSA, Trieste, Italy; 2 Cognitive Development Center, Central European University, Budapest, Hungary; 3 Department of Psychological Sciences, Birkbeck, University of London, London, United Kingdom; University of Portsmouth, UNITED KINGDOM

## Abstract

Observed associations between events can be validated by statistical information of reliability or by testament of communicative sources. We tested whether toddlers learn from their own observation of efficiency, assessed by statistical information on reliability of interventions, or from communicatively presented demonstration, when these two potential types of evidence of validity of interventions on a novel artifact are contrasted with each other. Eighteen-month-old infants observed two adults, one operating the artifact by a method that was more efficient (2/3 probability of success) than that of the other (1/3 probability of success). Compared to the Baseline condition, in which communicative signals were not employed, infants tended to choose the less reliable method to operate the artifact when this method was demonstrated in a communicative manner in the Experimental condition. This finding demonstrates that, in certain circumstances, communicative sanctioning of reliability may override statistical evidence for young learners. Such a bias can serve fast and efficient transmission of knowledge between generations.

## Introduction

Various kinds of learning processes involve establishing associations between types of events in the world. Forming and storing such an association on the basis of event tokens is useful only if the association reflects a stable underlying regularity, which is likely to extend into the future. Thus, learning requires the assessment of the validity of the new association to be acquired for generalization to future encounters of the same type of event.

Most theories of learning point to statistical evaluation of co-occurrences as a way to ensure the reliability of learned associations. Indeed, there is a growing body of evidence that infants and adults acquire new knowledge by statistically optimal evaluation of the information provided by the samples of these events [1]. The success of explaining human behavior in this manner in a large variety of contexts [2–3] led recently to the proposal that human learning can be best described in a statistical learning framework [4].

However, statistical reliability is not the only type of information that can warrant the validity of associations between co-occurring events. In particular, human communication has been suggested to be able to convey generic, hence generalizable, knowledge to others in single demonstrations, which do not allow statistical evaluation of reliability of regularity [5–6]. These demonstrations can be performed non-verbally, where the accompanying ostensive-communicative signals function to assure the recipient of the relevance and generalizability of the information demonstrated. For example, when 14-month-old infants had been shown an unusual and seemingly inefficient action to operate a novel object, they tended to imitate this action only when the demonstrator made eye contact with them and talked to them in infant-directed speech (both are ostensive signals,’ see [7]) during the demonstration, but not when such communicative signals were omitted [8].

Our aim with the present study was to test whether young learners preferentially rely on statistical or communicative validation to assess information about the use of a novel object, when both types of information are available. We decided to investigate this question in the domain of social learning about artifacts because evidence suggests that human infants expect human-made objects to have functions [9] and to exhibit a regular relation between interventions and effects [10]. It has been shown that, when infants learn how to operate a novel artifact, communicative demonstrations can overrule efficiency considerations [11–12]. Statistical information about the probability of a certain intervention producing an effect (i. e., its reliability) may also reflect the efficiency of the intervention: an action that is more reliably produce an effect is more efficient than another one because it requires less cost (fewer number of actions) to invest in operating the artifact. Thus, our study provided infants with information about the efficiency of two alternative methods of operating a novel artifact in terms of lower (1/3) or higher (2/3) probability of producing an effect, and we tested whether a communicative context, indicated by the presence of ostensive signals, could shift their choice of action from the statistically more reliable (efficient) toward the less reliable (inefficient) option.

## Materials and Methods

Infants were tested in two different conditions. In the Baseline Condition only statistical information was available to discriminate between two options to act, while in the Experimental condition this statistical information was pitted against ostensive signals that suggested that the less reliable response was to be preferred.

### Participants

Two groups of 18-month-old infants participated in the study. Forty infants were assigned to the Baseline Condition (23 females, mean age 18;6) and forty infants were assigned to the Experimental Condition (25 females, mean age 18;10). The parents of all participants provided written informed consent, and this study was approved by the Hungarian United Ethical Review Committee for Research in Psychology (EPKEB).

### Apparatus

**Fig 1:**
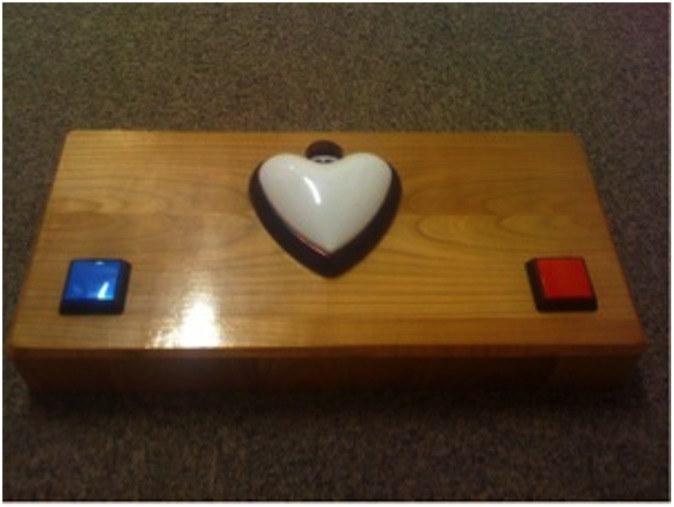
The experimental apparatus.

We used a custom-made 50 x 20 cm wooden experimental box with two differently colored buttons on the two sides of the box and a heart-shaped white lamp in the middle (Fig. 1). Both buttons turned the light on in the lamp and produced a bell sound at the same time. Two hidden switches on the back of the box could activate or deactivate the two buttons.

### Procedure

**Fig 2:**
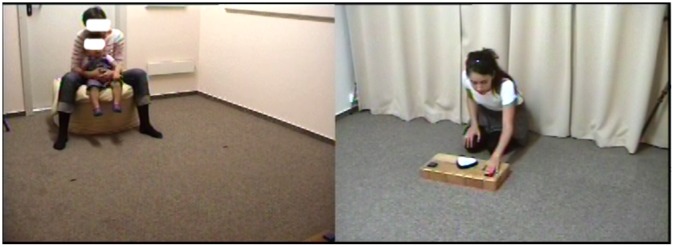
The experimental procedure. The infant sat in the lap of a parent facing the apparatus operated by experimenters.

The experiment consisted of a demonstration phase and a test phase. During the demonstration phase, infants sat in their parents’ lap on a bean bag 3 meters from the experimental apparatus, and observed two female experimenters (E1 and E2) operating it (Fig. 2). First E1 entered the room from behind a curtain behind the apparatus and pressed one of the buttons three times in a row. These actions did or did not produce light and sound effects, depending the state of the hidden switches, operated by the experimenter. Then she left behind a curtain, and E2 entered the room the same way. She pressed the other button of the apparatus three times, and then left the room. Immediately after this, the test phase started, during which the infants were allowed to approach the apparatus and explore it by pressing its buttons. This procedure was designed to test how the success and the nature of the two demonstrations, determined by the conditions (see below), influenced children’s first choice of buttons to approach. For each infant, one button was assigned to be the reliable (efficient) button, the other to be the unreliable (inefficient) button. The efficient button produced the light and the sound effect for the first two presses, but not for the third one, while the inefficient button produced an effect only for the first press. Note that neither button was completely reliable or unreliable: the difference was in the degree of reliability rather than a categorical one. The side of the reliable/unreliable button, and the order of demonstration (reliable first or unreliable first) were counterbalanced across infants.

#### Baseline Condition

In this condition, neither experimenter communicated with the infant. Thus, when they entered the room, they went straight to the apparatus, without greeting the infants or making eye contact with them. During the demonstration, they did not talk, and kept their gaze on the apparatus.

#### Experimental Condition

In this condition, the experimenter who performed the demonstration on the unreliable button did so in a communicative manner. When she entered the room, she greeted the infant, and said (in Hungarian), “Look, I am going to show you something very exciting!” After the first press, which produced a light as sound as an outcome, she reacted, “Look, isn’t it very exciting? Shall we do it again?” She then pressed the button two more times without an effect, on which she did not make any comment. The experimenter who operated the reliable button acted the same, non-communicative way as in the Baseline Condition. Note that the communicative demonstration was the first one for half of the infants in this condition, and it was the second one for the other half.

The single dependent variable we recorded was the first button that infants touched when they reached the apparatus.

## Results

**Fig 3:**
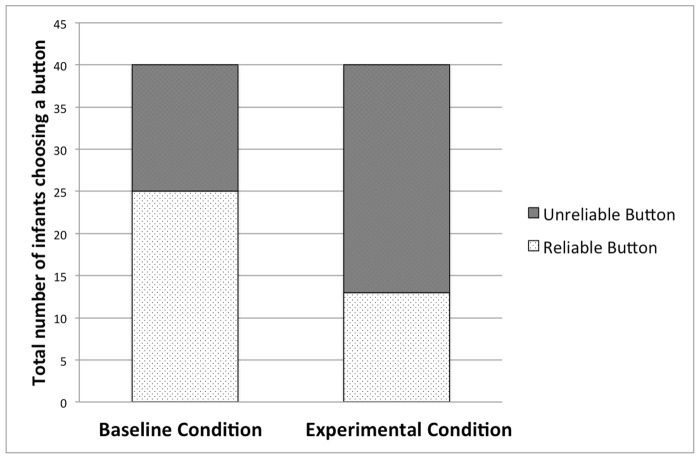
Results. Number of infants choosing the reliable and the unreliable button as a function of demonstration condition.

Fig. 3 depicts the number of infants choosing one or the other button in the two conditions. The main purpose of this study was to test whether ostensive signals could shift infants’ action preference from the reliable button towards the unreliable button. A Fisher’s exact test confirmed this prediction by showing that more children chose the unreliable button in the Experimental Condition than in the Baseline Condition (*p* = .013, two-tailed). While in the Baseline Condition infants did not necessarily choose the reliable button (25 of 40 infants did so), in the Experimental Condition a significant majority (27 of 40 infants, *p* = .038 by two-tailed binomial test, assuming equal probability as chance) opted for the unreliable button. We also tested whether the order of demonstrations had an effect on infants’ choice: 48 of 80 infants touched first the button that had been demonstrated second, which is not different from chance level (*p* = .093 by binomial test).

## Discussion and Conclusion

When two sources of information were contrasted with each other, infants seem to have trusted more the communicator than their own eyes. Although they did not show strong evidence of relying statistical information in this study, further findings suggest that, when other cues are held constant, young children can learn from the observation of probability of success of different interventions, and can use this information for selecting their own actions [13]. Our results indicate that statistical evidence, which tends to govern individual learning and observational social learning, may be easily overridden by ostensive signals that directly indicate the relevance of demonstrated information for toddlers. Whether such overreliance on others in learning is restricted to domains, such as the function and use of human-made artifacts, in which the to-be-acquired knowledge is inherently cultural [14], is a question for further research.

A further question concerns the mechanism by which communicative signals induced preference for the unreliable button. Our hypothesis was that these signals would directly indicate to children an opportunity for learning about the referent artefact, and their choice of buttons would be influenced by the knowledge they acquired from the ostensive demonstration. Alternatively, it is also possible that communicative signals simply highlighted, or associated with, one of the buttons, and this effect directly influenced children’s preference, without making them learn anything new. Although the present study is unable to discriminate between these options to reveal the underlying cognitive mechanisms, our results show that communicative signals can be more powerful than statistical reliability in modulating infants’ actions on novel artefacts. This effect is predicted by the theoretical framework that yielded the hypothesis of the present study [6], but further studies are needed to clarify the exact mechanisms by which ostensive signals influence infants’ re-enactment of adults’ actions.

While our finding suggests that communicative information can, in some circumstances, trump statistical information, we do not propose that this will always be the case. An obvious difference between these two sources of information lies in their frequency dependence. Communicative demonstrations, on the one hand, are assumed to indicate the validity of the demonstrated association without any repetition [5]. On the other hand, perhaps three samples per option provided infants with only weak statistical evidence of reliability difference between the buttons in our study, and longer demonstrations would change their preference to the more reliable one even against communicative signals. Nevertheless, our study illustrates that statistical evidence is not the only, and perhaps not even the most important, source of information supporting the acquisition of knowledge in human infants and toddlers. The bias towards favoring communicatively conveyed information can serve as the basis of fast and efficient transmission of knowledge between individuals [15].

## Supporting Information

S1 Dataset The data collected from the Experimental Group. (XLSX)

S2 Dataset The data collected from the Control Group. (XLSX)
